# Low level of perfectionism as a possible risk factor for suicide in adolescents with attention-deficit/hyperactivity disorder

**DOI:** 10.1192/j.eurpsy.2022.646

**Published:** 2022-09-01

**Authors:** L. Katzenmajer-Pump, B. Farkas, B. Varga, J. Jansma, J. Balazs

**Affiliations:** 1 Institute of Psychology, Eötvös Loránd University, Doctoral School Of Psychology, Budapest, Hungary; 2 Semmelweis University, Doctoral School Of Mental Health Sciences, Budapest, Hungary; 3 UMC Utrecht, Brain Center Rudolf Magnus, Department Of Neurology And Neurosurgery, Utrecht, Netherlands; 4 Eotvos Lorant University, Developmental And Clinical Child Psychology, Budapest, Hungary

**Keywords:** Suicide, adhd, perfectionism, riskfactor

## Abstract

**Introduction:**

Previous research highlighted that adolescents with attention-deficit/hyperactivity disorder (ADHD) are four times as vulnerable to suicidal behavior as the healthy population. Maladaptive perfectionism is also viewed as an important risk factor for suicide. Yet, there are no studies which focused on the relationship between perfectionism and suicide among adolescents with ADHD.

**Objectives:**

The objective of the present study was to explore if perfectionism may be a risk factor for suicidal behavior in adolescents with ADHD.

**Methods:**

The clinical group was recruited from outpatient clinics, while the non-clinical group was recruited from high schools around Hungary. The clinical group’s inclusion criterion was ADHD diagnoses, while the non-clinical group required the absence of any current or past psychiatric treatment or diagnoses.

**Results:**

In the ADHD group 88 adolescents participated, and 96 adolescents participated in the non-clinical group. There was no difference regarding the level of perfectionism in the groups, except one dimension of perfectionism, which is ‘Organization’. The ADHD group had significantly higher level of suicidal behavior than the control group ((χ2 (1) = 11.222, p < .001, V = 0.25). Among the ADHD group adaptive perfectionism was significantly negatively correlated with suicidal behaviour.

**Conclusions:**

Adolescents with ADHD did not have a different level of perfectionism than the healthy control group only in ‘Organization’ trait. This result could add to the therapeutic work with adolescents diagnosed with ADHD with underlining the importance to focus on organizational skills. The result highlights that adaptive perfectionism appears to be a protective factor against suicidality.

**Disclosure:**

No significant relationships.

